# Effects of anabaena lipoxygenase on whole wheat dough properties and bread quality

**DOI:** 10.1002/fsn3.1782

**Published:** 2020-08-28

**Authors:** Kexin Shi, Pei Wang, Chong Zhang, Zhaoxin Lu, Meirong Chen, Fengxia Lu

**Affiliations:** ^1^ College of Food Science and Technology Nanjing Agriculture University Nanjing China

**Keywords:** lipoxygenase, processing quality, rheological properties, whole wheat flour

## Abstract

The effects of the purified recombinant anabaena lipoxygenase (ana‐rLOX) on the rheological characteristics of whole wheat dough and the quality of bread were investigated. The lightness of whole wheat dough supplemented with ana‐rLOX was improved, which is superior to that of dough treated with benzoyl peroxide. The effect of ana‐rLOX on the strength of dough was analyzed by farinograph, extensograph, and dynamic rheological tests. Compared with the control, the stability time of dough treated with 40 IU/g ana‐rLOX increased by 35.4% and the farinograph quality number increased by 27.4%. In addition, the resistance to extension, as well as the elastic and viscous modulus, was improved by ana‐rLOX in a dose‐dependent manner. The height and specific volume of bread treated with ana‐rLOX increased by 17.3 and 15.2%, respectively, compared with the control, and the lightless, whiteness, and other textural parameters, such as hardness, springiness, chewiness, resilience, and gumminess, were significantly improved. Overall, the results of this study suggest the promising application of ana‐rLOX in enhancing quality of whole wheat flour.

## Graphical abstract

The effects of purified recombinant anabaena lipoxygenase (ana‐rLOX) on the rheological properties of whole wheat dough and the quality of bread were investigated.

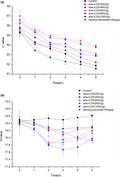

## 1 INTRODUCTION

Whole wheat flour belongs to the whole grain category and is made from whole wheat, in which all components, including bran, germ, and endosperm, are present in the same relative proportions of the intact grain (Niu, Hou, Kindelspire, Krishnan, & Zhao, 2017). Due to their unique flavor and enriched functional nutrients such as vitamins, dietary fiber, minerals, enzymes, and phenolic compounds (Hirawan, Ser, Arntfield, & Beta, 2010), whole wheat flour and its products have been popular and increasingly marketed in recent years. The regular consumption of whole grain products can provide health benefits such as the reduced risk of obesity, cerebrovascular disease, and cardiovascular disease (Giacco, Della Pepa, Luongo, & Riccardi, 2011). However, the presence of bran in whole wheat flour causes a dilution of gluten proteins, resulting in the poor processing characteristics (Jiang, Martin, Okot‐Kotber, & Seib, 2011) and reduced organoleptic characteristics of the final products (Yang et al., 2014). To overcome these drawbacks, some chemical agents were used to enhance the quality of wheat flour, such as benzoyl peroxide and potassium bromate (KBrO_3_), which, however, could impose side effect to human health (Kujawska et al., 2013). Therefore, researchers are currently looking for effective and safe wheat flour additives. Enzyme has recently attracted widespread attention as an alternative additive for whole wheat flour (Liu et al., 2018). Specifically, α‐amylase, lipase, glucose oxidase (GOX), transglutaminase, and xylanase have been reported to modify the elasticity of gluten, enhance the rheological characteristics of whole wheat dough, and increase the specific volume of bread (Geng, Harnly, & Chen, 2016; Ghoshal, Shivhare, & Banerjee, 2013; Steffolani, Ribotta, Perez, & Leon, 2012; Tang et al., 2014).

Lipoxygenase (LOX) catalyzes the regioselective and stereoselective dioxygenation of polyunsaturated fatty acids (PUFAs) into hydroperoxides, which are subsequently converted into lipophilic pigments and thiol groups (Casey et al., 1999). LOX has been widely used in food processing to blench flour and enhance the strength of gluten (Addo, Burton, Stuart, Burton, & Hildebrand, 2006; Ribotta, Pérez, Añón, & León, 2010). Furthermore, the addition of soybean powder in the breadmaking process has been proved to enhance dough rheology and bread volume (Junqueira, Cocato, Colli, & Castro, 2008). Commercial LOX is extracted mainly from plant tissues such as soybean powder, which typically contains a mixture of enzymes that may reduce the catalyzing effects of LOX (Junqueira et al., 2008). The better way to obtain purified lipoxygenase is using heterologous expression system to produce the enzyme by the means of molecule cloning (Casey et al., 1999). In our previous report, the LOX gene from *Anabaena* sp. PCC 7,120 was successfully expressed in *Bacillus subtilis* extracellularly (Zhang et al., 2012). And the purified recombinant lipoxygenase was able to promote bleaching and fortify gluten quality (Yang Mai 13 variety) (Zhang et al., 2013).

In this study, another wheat variety, Yang Mai 16 with a medium gluten content, was used to evaluate the ability of ana‐rLOX to improve whole wheat dough and bread processing properties by rheological and textural profile analysis, respectively. To the best of our knowledge, this is the first report on the use of recombinant lipoxygenase in whole wheat flour.

## 2 MATERIALS AND METHODS

### 2.1 Preparation of ana‐rLOX, whole wheat flour, and other materials

The production of recombinant lipoxygenase and the assay of its enzymatic activity were performed as previously reported (Zhang et al., 2013).

Freshly milled whole wheat flour was obtained from Yang Mai 16 (harvested in 2018, Jiangsu, China). The cleaned wheat was grounded with a Buhler laboratory mill (Buhler, Switzerland), and the bran was separated from the flour using a sifter. The separated bran was treated in high pressure sterilizer at 105°C for 10 min. Subsequently, the bran was grounded by high‐speed pulverizer (Dade, Zhejiang, China) to pass through an 80‐mesh sieve and then added back to the wheat flour at the initial proportion. After preparation, the unbleached whole wheat flour without chemical or enzyme additives was stored in self‐sealed bags at 25°C. The whole wheat flour properties were determined according to national standards (GB 5009.3‐2016; GB 5009.5‐2016; GB 5009.4‐2016), with 13.2% moisture, 12.1% protein, and 1.3% ash, and no endogenous LOX activity.

Linoleic acid (L1376‐1G) was purchased from Sigma‐Aldrich, and instant dry yeast (Angel Yeast Co.) was purchased from the local market. All chemicals used in this study were analytical grade.

### 2.2 Whole wheat dough preparation

The dough was prepared in a Farinograph (Brabender) using the 10‐g mixing bowl with the whole wheat flour. Potassium bromate (KBrO_3_, 50 μg/g), benzoyl peroxide (150 μg/g), and different levels of ana‐rLOX (20, 40, 60, 80 and 100 IU/g) were applied to the whole wheat flour for different dough preparation.

### 2.3 Application of ana‐rLOX in dough and its color measurements

After the dough was molded, it was kept in transparent plastic bags to prevent water evaporation. The dough was stored at room temperature for 5 hr and sampled every half hour. A color difference meter (Konika Minolta) was used to evaluate the changes in dough color using the Hunter color manual, where the color of a sample is denoted by the three dimensions, including L*, a*, and b* values. The dough was placed in a cabinet maintained 85% relative humidity at 25°C between tests.

### 2.4 Farinograph analysis

The farinograph properties of whole wheat flour were determined using a Farinograph according to the AACC method 54‐21. The whole wheat flour, adjusted moisture to 14%, was added into a mixing bowl and then mixed for 1 min. The maximum dough consistency should be within the range of 500 ± 20 BU, and the total mixing time was 20 min. The water absorption rate of the dough, development and stabilization time of dough, degree of softening, and the farinograph quality number were determined.

### 2.5 Extensograph analysis

The dough extensible parameters were measured with a TA‐XT2 Texture Analyser (Stable Micro Systems,). The dough was pressed into a 2 mm × 60 mm dough strip using a Teflon plate and then stored at 25°C for 30 min. Subsequently, the dough strip was subjected to a tensile test using a tensile probe (SME A/KIE) until the dough strip broke. The maximum resistance to extension (R, g), extensibility (E, mm), and area under the curve (g mm) of the whole wheat dough were measured by obtaining a resistance to extension‐extensibility curve.

### 2.6 Dynamic rheological measurement

The dynamic rheological characteristics of the samples with different treatments were analyzed using an AR1000 rheometer (TA Instruments Ltd., Crawley, UK) according to a previously reported method (Zhang et al., 2012). The diameter and gap distance of the steel plate were 40 mm and 2.5 mm, respectively. The whole wheat dough samples were immediately removed, sealed with plastic wrap, and stored at 25°C for 1 hr. Then, the settled dough was placed on the test bench, and paraffin was used to prevent the samples from drying during the measurement. Prior to the tests, doughs were spread for 10 min to relax the residual strain. All the tests were performed at 25°C. The elastic and viscous modulus were evaluated based on the frequency sweep tests from 0.01 to 20 Hz. The measurements were performed three times for each sample.

### 2.7 Analysis of baking and bread properties

#### 2.7.1 Breadmaking procedure

The process of bread manufacturing was performed according to the method recorded in the reported literature (Zhang et al., 2013) with some modifications. The whole wheat bread ingredients included whole wheat flour (150 g), water (85 g), dry yeast (3 g), sugar (10 g), and salt (2 g). Different whole wheat breads were prepared by adding different additives (benzoyl peroxide, KBrO_3_, and different levels of ana‐rLOX), respectively.

All ingredients were added to a needle mixer and mixed for 10 min at the speed of 120 rpm. The dough was then kept for fermentation in a cabinet at 30°C and 85% relative humidity for 1 hr, after which the dough was divided into three pieces of equal weight. After being pressed several times through the tablet machine, the dough was stored for 20 min before being rounded and formed. Finally, the dough was fermented twice in a humidity chamber for 50 min and then baked at 180°C for half an hour in a baking oven.

#### 2.7.2 Specific volume measurement

After the whole wheat bread was cooled at room temperature for 2 hr, it was weighed and analyzed for quality. The volume was examined according to rapeseed displacement method, and the specific volume (SV, cm^3^/g) was evaluated as the ratio of the volume and the mass of the bread: specific volume = volume (cm^3^)/ mass (g).

#### 2.7.3 Crumb color measurement

The whole wheat bread was cut into several 20 mm slices, and then, the crumb color at three different locations of one slice, including the L* (lightness), a* (redness/greenness), and b* (yellowness/blueness) values, was determined using a color difference meter. Each sample was measured five times. The whiteness was calculated as the following equation: Hunter value (Wh)= 100‐ [(100‐ L *)^2^ + a * ^2^ + b *^2^]^1/2^.

#### 2.7.4 Textural profile analysis (TPA)

TPA of the whole wheat bread crumb was performed using a TA‐XT2 Texture Analyser equipped with a P/50 probe. The maximum deformation was 40% of the bread height and the interval time was 30 s. The pretest speed, test speed, and post‐test speed were 1, 1 and 10 mm/s, respectively. According to the force–distance graph, the hardness, elasticity, adhesion, and chewiness were determined, and the averages of five tests were calculated.

### 2.8 Statistical analysis

Analysis of variance (ANOVA) of data was analyzed with the SPSS 17.0 (SPSS Institute, USA). The mean values were compared using the Duncan's multiple range tests with a significance level of *p* ≤ .05.

## 3 RESULTS AND DISCUSSION

### 3.1 Bleaching effect of ana‐rLOX on whole wheat dough

**FIGURE 1:**
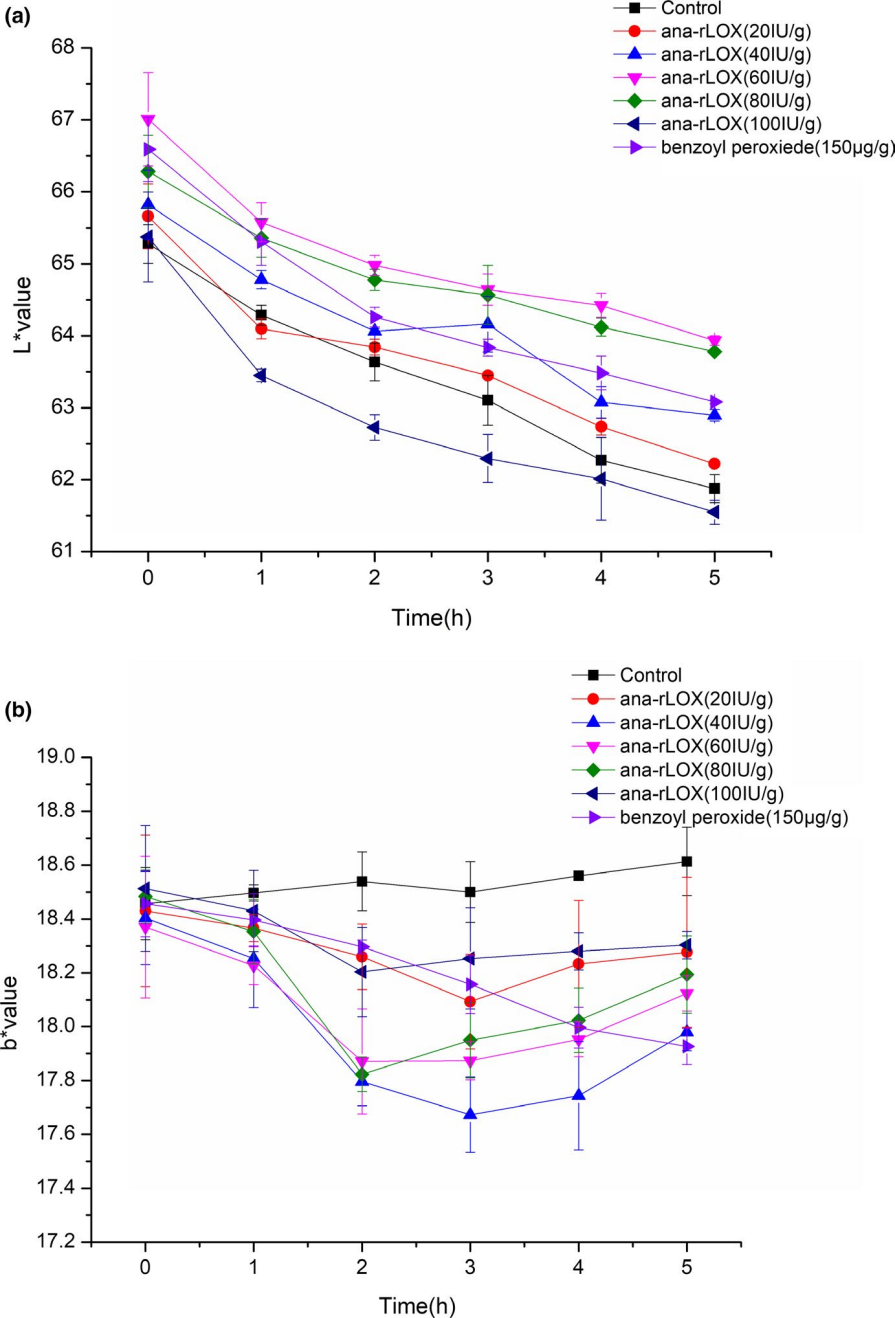
Changes in color components including L* (a), 100 for white, 0 for black) and b* (b),﹢for yellow, ‐ for blue) values of whole wheat dough with different additives at 25°C

The L* value is an indicator of the lightness of the product, from 0 for black to 100 for perfect white, and the dough with high lightness is associated with a high final production quality. When treated with ana‐rLOX, the L* value of whole wheat dough was higher than the control, and the whole wheat dough treated with 60 IU/g ana‐rLOX showed the highest L* value (Figure 1a). The yellowness (b* value) reflects the carotenoid content, an organic pigment widely present in grain and directly associated with the color of cereal grain products (Yang et al., 2014). The b* value of the ana‐rLOX added whole wheat dough decreased during the first 3 hr (Figure 1b), indicating that ana‐rLOX could reduce the carotenoid content, which is consistent with the previous research (Hidalgo, Brandolini, & Pompei, 2010). As expected, the L* value of the dough supplemented with benzoyl peroxide decreased compared with the control, because this component could promote oxidation of carotenoid via a typical free radical mechanism (Lamsala & Faubion, 2009). Ana‐rLOX, as a fast oxidant, can quickly play a role in the short term, and it can promote flour maturation and improve whiteness. However, with increasing time, the b* value gradually rebounded, which may due to the decreased activity of ana‐rLOX over time and a prominent role of polyphenol oxidase present in dough.

### 3.2 Effect of ana‐rLOX on the rheological properties of whole wheat dough

#### 3.2.1 Farinograph test results

Industrial whole wheat flour should have favorable rheological characteristic to ensure the end‐product quality (Armero and Collar, 1998; Bueno, Thys, & Rodrigues, 2016). With ana‐rLOX addition, water absorption of whole wheat flour, and development and stabilization time of dough improved, while the degree of softening declined, together giving a higher farinograph quality number than that of the control (Table 1). When the whole wheat dough was supplemented with 40 IU/g ana‐rLOX, the dough formation time and stability time were increased by 2.3 and 2.8 min, respectively, while the degree of softening decreased by 9 units and the farinograph quality number increased by 32 units, compared with that of the control. The stabilization time, which reflects the stability and toughness of the dough (Zhang et al., 2012), significantly increased when the whole wheat flour was supplemented with ana‐rLOX, indicating enhanced dough stability and maximum resistance to mixing. Our results are consistent with the point that LOX could provide molecular oxygen to polyunsaturated fatty acids (Huang, Hua, & Qiu, 2006), which is accompanied by the formation of the inter‐ and intramolecular disulfide bonds between gluten proteins (Zhang et al., 2016).

**TABLE 1:** Different levels of ana‐rLOX affect farinograph properties of whole wheat flou

Samples	Water absorption (%)	Development time (min)	Stabilization time(min)	Degree of softening (FU)	Farinograph quality number
Control	62.6 ± 0.6a	8.0 ± 0.6a	7.9 ± 0.2ab	15 ± 1.2d	117 ± 1.7a
KBrO_3_ (50μg/g)	63.5 ± 0.4bc	8.3 ± 0.1ab	8.2 ± 0.06b	13 ± 0.6d	123 ± 1.2bc
Ana‐rLOX (20 IU/g)	62.9 ± 0.06ab	8.2 ± 0.1ab	7.7 ± 0.1a	9 ± 0.6b	122 ± 0.6b
Ana‐rLOX (40 IU/g)	63.5 ± 0.5bc	10.3 ± 0.7d	10.7 ± 0.5e	6 ± 0.6a	149 ± 2.1f
Ana‐rLOX (60 IU/g)	63.3 ± 0.6b	9.2 ± 0.2c	9.5 ± 0.06d	5 ± 0.6a	142 ± 1.5e
Ana‐rLOX (80 IU/g)	63.5 ± 0.06bc	8.6 ± 0.2b	8.7 ± 0.06c	6 ± 1.2a	135 ± 1.9d
Ana‐rLOX (100 IU/g)	63.6 ± 0.1c	8.4 ± 0.06ab	7.8 ± 0.1a	11 ± 0.6c	126 ± 1.2c

Note

Data expressed as means ± standard. Values followed by the different letters in the same column are statistically different (*p* < .05).

KBrO_3_ is a slow‐acting oxidant (Junqueira et al., 2007) that plays an important role in bread proofing and baking (Lagrain, Thewissen, Brijs, & Delcour, 2007). In comparison, enzyme preparations can work quickly as an efficient oxidant during dough making. An investigation of the amount effect of ana‐rLOX on the whole wheat flour indicated that the dough quality will not be further enhanced when the level of ana‐rLOX was higher than 40 IU/g, which may owe to the excessive oxidation of gluten caused by excessive ana‐rLOX (Niu et al., 2017).

#### 3.2.2 Extensograph test results

Dough modulation is the basis for preparing final products (Nie et al., 2019). The extensographic properties of the whole wheat dough supplemented with ana‐rLOX were improved, especially resistance to extension which increased (Table 2). Resistance to extension reflects the elasticity of the dough. In our study, the treatment of ana‐rLOX significantly improved the maximum resistance to extension of the whole wheat dough. The 40 IU/g ana‐rLOX added dough exhibited greater improvement than the dough treated with KBrO_3_, and the resistance to extension was increased by 19.9 and 9.8% compared with the control and potassium bromate groups, while the area under the curve increased by 24.9 and 6.3%, respectively. These results suggested that ana‐rLOX could remarkably improve the strength of the whole wheat dough .

**TABLE 2:** Different levels of ana‐rLOX affect extensograph properties of whole wheat flour

Samples	Resistance to extension (g)	Extensibility (mm)	Area under the curve (g•mm)	Ratio R/E
Control	22.96 ± 0.28a	32.22 ± 0.38a	91.04 ± 0.63a	0.713
KBrO_3_ (50 μg/g)	25.09 ± 0.23b	31.83 ± 0.56a	106.95 ± 0.67b	0.788
Ana‐rLOX (20 IU/g)	23.51 ± 0.29c	31.55 ± 0.40a	102.71 ± 0.97c	0.745
Ana‐rLOX (40 IU/g)	27.54 ± 0.16d	31.94 ± 0.54a	113.67 ± 0.44d	0.862
Ana‐rLOX (60 IU/g)	25.93 ± 0.21e	30.67 ± 0.05b	107.40 ± 0.18c	0.845
Ana‐rLOX (80 IU/g)	24.20 ± 0.35f	30.59 ± 0.28b	105.73 ± 0.51e	0.791
Ana‐rLOX (100 IU/g)	22.83 ± 0.18a	29.16 ± 0.09c	102.76 ± 0.42b	0.783

Note

Data expressed as means ± standard. Values followed by the different letters in the same column are significantly different (*p* < .05).

#### 3.2.3 Dynamic rheological measurements results

**FIGURE 2:**
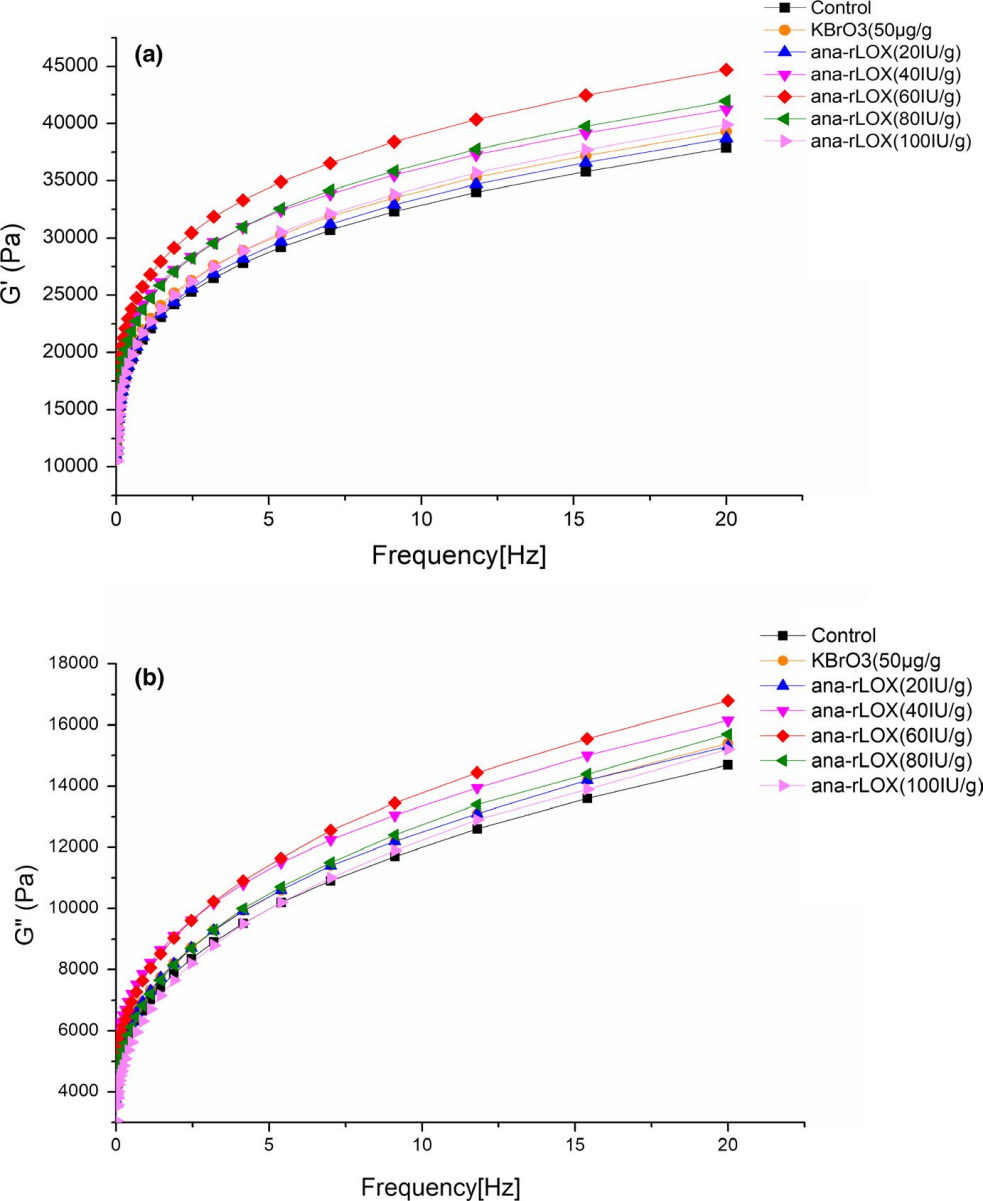
Effect of different addition levels of ana‐rLOX on the elastic modulus G' (a) and viscous modulus G'' (b) of whole wheat dough

The changes of viscoelasticity of the whole wheat dough were analyzed by comparing the elastic and viscous modulus. High G' and G'' values indicate a better elasticity and extensibility of dough and thus resulted in the increased expansion of whole‐wheat baking products (Wang, Huang, Kim, Liu, & Tilley, 2011). Both the G' and G'' values of the sample treated with KBrO_3_ and ana‐rLOX increased compared with those of the control (Figure 2), consistent with the previous result that the gluten network in the dough was improved by the catalysis of lipoxygenase (Zhang et al., 2016). Both the elastic and viscous modulus of the dough enhanced with the higher concentrations of ana‐rLOX, and when treated with 60 IU/g of ana‐rLOX, the G' value increased by 17.9% compared with the control, indicating that the treatment of ana‐rLOX enhanced ability of the dough to restore its original shape after deformation (Han et al., 2013; Li, Hou, Chen, & Gehring, 2013).

### 3.3 Ana‐rLOX improves the quality of whole wheat bread

#### 3.3.1 Bread volume

**FIGURE 3:**
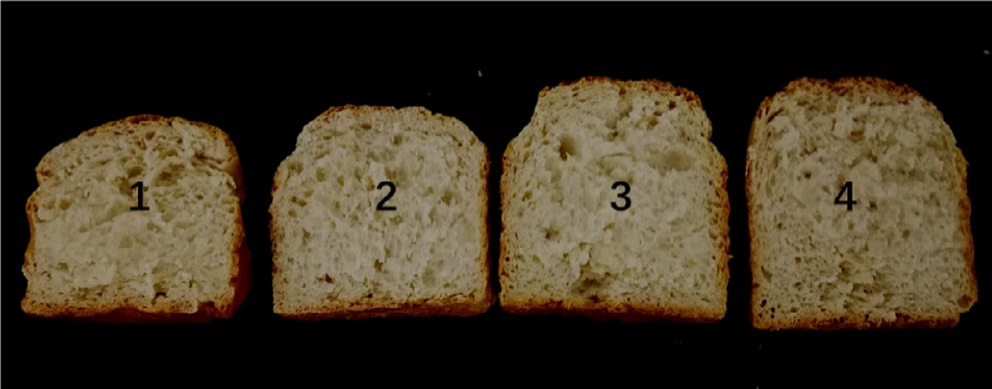
Whole wheat bread slice photographs obtained from specified experiments. (1) control; (2) added with 50 μg/g KBrO_3_; (3) added with 40 IU/g ana‐rLOX; and (4) added with 60 IU/g ana‐rLOX

The overall effects of the ana‐rLOX treatment can be concluded by the appearance of the whole wheat bread slices (Figure 3). The bread slice diagram showed that the whole wheat bread treated with ana‐rLOX was larger than that of the control and the bread treated with KBrO_3_. The height, volume, and specific volume of the different breads are listed in Table 3. Addition of ana‐rLOX contributed to the improvement in loaf volume. When 40 IU/g ana‐rLOX was added, the loaf height and volume of whole wheat bread increased by 17.3 and 15.7%, respectively, resulted in the specific volume increased 15.2%, compared with the control.

**TABLE 3:** Physical properties of whole wheat bread

Samples	Loaf weight (g)	Bake volume (cm^3^)	Height (cm)	Specific volume (cm^3^/g)
Control	269.47 ± 0.30a	773.05 ± 3.07a	5.95 ± 0.17a	2.869 ± 0.01a
KBrO_3_ (50 μg/g)	271.59 ± 0.09a	822.46 ± 1.36b	6.50 ± 0.11b	3.028 ± 0.01b
Ana‐rLOX (40 IU/g)	270.61 ± 0.53a	894.97 ± 7.59c	6.98 ± 0.18c	3.304 ± 0.03c
Ana‐rLOX (60 IU/g)	271.20 ± 0.16a	851.85 ± 5.71d	6.88 ± 0.17c	3.208 ± 0.01c

Note

Data expressed as means ± standard. Values of the same column followed by the different letters are statistically different (*p* < .05).

#### 3.3.2 Crumb color

Currently, poor appearance of flour products is a major problem of whole wheat foods. Therefore, it is of great significance to improve the color of whole wheat products. The crumb color of the different groups is shown in Table 4. LOX, which needs water and oxygen to become active, plays an important role in bread making, as compared with benzoyl peroxide (Matsushita et al., 2017). It was reported that when treated with benzoyl peroxide, the b* value of crumb decreased significantly, while the L* value just changed slightly, which may be related to the ability of benzoyl peroxide to degrade carotenoid in the flour (Hidalgo et al., 2010). By contrast, in our study, ana‐rLOX has a positive effect on bread color. Compared with that of the control, the addition of ana‐rLOX makes whole wheat bread brighter, with an increase of 3.2 units of L* value when added with 40 IU/g ana‐rLOX. Furthermore, the a* value which represents the redness (Li, Wang, & Krishnan, 2020) was decreased compared with that of the control. The high bran content of whole wheat flour causes the low whiteness of the bread. When treated with ana‐rLOX, the whiteness (Hunter value) of the bread increased significantly (*p* < .05), which was 3.26 units higher than those of the control.

**TABLE 4:** The color of whole wheat breads supplemented with different processes

Samples	L*	a*	b*	Hunter Value (Wh)
Control	58.25 ± 0.60a	1.83 ± 0.04b	17.64 ± 0.31c	54.64 ± 0.08a
Benzoyl peroxide (150 μg/g)	59.06 ± 0.29b	1.71 ± 0.11b	16.56 ± 0.12a	55.81 ± 0.02b
Ana‐rLOX (40 IU/g)	61.45 ± 0.07c	1.39 ± 0.09a	16.87 ± 0.15ab	57.90 ± 0.01d
Ana‐rLOX (60 IU/g)	60.62 ± 0.12d	1.48 ± 0.12a	17.15 ± 0.11b	57.02 ± 0.03c

Note

Data expressed as means ± standard. Values followed by the different letters in the same column are statistically different (*p* < .05).

#### 3.3.3 Bread texture

**FIGURE 4:**
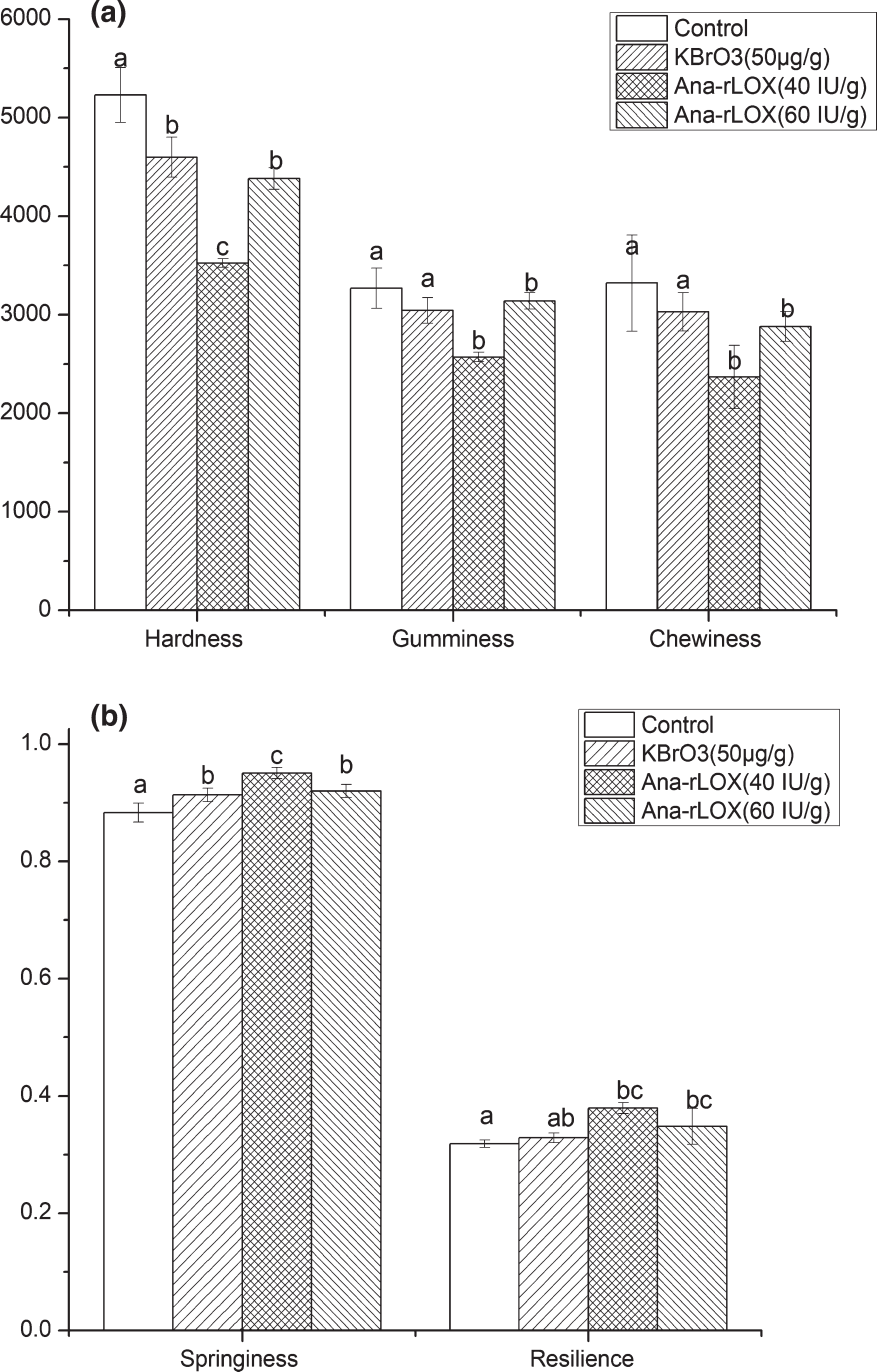
Textural analysis of whole wheat bread

It was reported that there are correlations between bread quality and hardness, resilience, springiness, and chewiness (Junqueira et al., 2007). In our study, whole wheat bread supplemented with ana‐rLOX was softer compared with the other samples, with hardness 32.6 and 23.3% lower than that of the control and KBrO_3_ added samples, respectively (Figure 4). In addition, the springiness of enzyme‐treated bread was enhanced. However, the effect of ana‐rLOX diminished at higher ana‐rLOX levels (60 IU/g). The results showed that the enzyme‐treated whole wheat bread got softer and the quality of the food was improved. Overall, the addition of ana‐rLOX in whole wheat flour could strengthen the gluten proteins structure, which resulted in an increased specific volume and a softer texture of bread, ultimately leading to a better springiness in the final product.

## 4 CONCLUSION

In this study, by ana‐rLOX treatment, the whiteness and rheological properties of whole‐wheat dough and the organoleptic quality of the whole wheat bread were improved. Ana‐rLOX reinforced the gluten network structure, and it led to decreases in rheological characteristics of whole wheat dough and improve the properties of whole wheat bread. Furthermore, these colligative effects were associated with the amount of enzyme added. The results of this study show that ana‐rLOX may replace chemical modifiers to improve the quality of whole wheat flour and could provide a scientific foundation for application of ana‐rLOX in food industry.
